# The Illegal Catch-and-Release of Wallabies

**DOI:** 10.3390/ani15182700

**Published:** 2025-09-15

**Authors:** Geoff Kaine, Vic Wright

**Affiliations:** 1 Manaaki Whenua—Landcare Research, Hamilton 2340, New Zealand; 2 UNE Business School, University of New England, Armidale, NSW 2351, Australia; vwright5@une.edu.au

**Keywords:** pest control, eradication, wallabies, translocation, catch-and-release, illegal behaviour

## Abstract

Internationally, introduced invasive species inflict incalculable damage on native species and primary production. In New Zealand, wallabies, which were introduced from Australia, are damaging the native and agricultural environments, and their range is spreading. As a first step towards the long-term goal of eradicating wallabies from New Zealand, the Tipu Mātoro National Wallaby Eradication Programme has the immediate goal of restricting wallabies to containment areas designated in regional pest management plans and to eliminate outlier populations by 2025. The success of this strategy is put at risk if people catch wallabies and release them outside the containment areas. We sought to characterise the motivations, beliefs, and attitudes of members of the public who might engage in this illegal behaviour by surveying members of the New Zealand public. We discovered that a very small proportion of the public supported the illegal catch-and-release of wallabies. Respondents, including hunters, that supported catch-and-release believed that wallabies create economic benefits and do not severely damage the environment. They also believed that hunting helps control wallaby numbers. These results yielded a variety of specific policy implications required to reduce catch-and-release behaviour.

## Simple Summary

In New Zealand, wallabies (*Notomacropus* spp.) have damaged the native and agricultural environments, and their range is spreading. Efforts to control them are being put at risk by people illegally catching-and-releasing wallabies outside current containment areas. We sought to characterise the motivations of members of the public who might catch-and-release wallabies with a view to reducing this illegal behaviour. We found that 6 per cent of participants in a national survey of New Zealand residents supported catch-and-release. It seems that their support for the release of wallabies outside containment areas is partly motivated by a belief that hunting would be easier and safer if wallabies were more widespread. These results highlight how vulnerable pest eradication programs can be to opposition from a very small group of people.

## 1. Introduction

Internationally, introduced invasive species inflict incalculable damage on native species and primary production [1]. In New Zealand, Bennett’s (or red-necked) wallabies (*Notomacropus rufogriseus*); Tammar (or Dama) wallabies (*Notomacropus eugenii*); and Parma wallabies (*Notamacropus parma*), which were introduced from Australia, are damaging the native and agricultural environments, and their range is spreading [2]. As a first step towards the long-term goal of eradicating wallabies from New Zealand, the Tipu Mātoro National Wallaby Eradication Programme has the immediate goal of restricting wallabies to containment areas designated in regional pest management plans and to eliminate outlier populations by 2025 [2]. Note that Parma wallabies are also present on Kawau Island but are not included in the National Wallaby Eradication Programme. The Programme is at risk because wallabies are being illegally caught and released outside the containment areas.

Historically, people have deliberately introduced invasive species for a variety of reasons [3,4]. Of these, a desire to improve recreational opportunities, to improve economic productivity, and/or to ensure animal welfare seem the most likely reasons for the illegal catch-and-release of invasive species today [4]. However, improving the aesthetics of the environment may still occasionally motivate the deliberate introduction of invasive species. The introduction and spread of lupins to New Zealand is a recent example [5].

There are numerous studies of illegal hunting or fishing (see [6,7,8,9] for examples). While illegal catch-and-release has long been recognised as a key factor in the spread of invasive species, there are few detailed studies on people’s reasons for engaging in illegal catch-and-release. These few studies [10,11,12] have mostly investigated reasons for illegally releasing, or illegally catching-and-releasing, sporting fish and game animals.

The illegal release of invasive species, such as disposing of surplus live bait and unwanted aquarium fish by releasing them into waterways, appears to be mainly motivated by convenience [10,13]. The illegal catch-and-release of invasive game species, such as wild pigs, is primarily motivated by a desire to improve opportunities for commercial or recreational hunting and associated commercial opportunities [12]. The illegal catch-and-release of invasive farmed species, such as mink and rabbits, appears to be mainly motivated by a desire to promote animal welfare [14].

In principle, the illegal catch-and-release of wallabies would thus seem most likely to be motivated by convenience or by a desire to improve opportunities for commercial or recreational hunting. Convenience might apply with respect to releasing individual wallabies that have been raised as pets and, as they have matured, become increasingly difficult to house and control. Hunters are the most likely source of pet wallabies as wallabies are particularly difficult to catch due to being cautious and nocturnal, preferring to rest in dense vegetation during the day and only emerging at dawn and dusk to graze the margins of open areas, like pastures and clearings [15,16]. We assume hand-raised wallabies have a low chance of survival to breed in the wild. As wallabies are hunted in New Zealand for recreation, catch-and-release may be motivated by a desire to improve hunting opportunities. As wallabies are not farmed in New Zealand, a desire to promote the welfare of farmed animals seems unlikely to motivate illegal catch-and-release.

Whatever the explanation for the introduction of wallabies into New Zealand, their spread has increased their negative impact on the environment [2]. Reducing this threat to sustainability will plainly require policy to modify relevant human behaviour. Prominent amongst this is the (illegal) catch-and-release of wallabies. To the best of our knowledge this is the first quantitative study of this behaviour and so contributes to the limited literature on the catch-and-release of invasive species and contemplation of possible policy responses.

## 2. Materials and Methods

We sought to characterise people’s reasons for illegal catch-and-release of wallabies through a national survey of the New Zealand public (n = 1000). Since catch-and-release is illegal, we could not, ethically, question people directly about this behaviour. We would also, anyway, expect that people’s responses to direct questions would be subject to social desirability bias [17]. Consequently, we designed a questionnaire that focused primarily on respondent beliefs, attitudes, and interest in relation to the eradication of wallabies, which is government policy. We hypothesised that the respondents’ support for illegal catch-and-release would be strongly negatively correlated with their support for wallaby eradication. We tested this hypothesis by including questions concerning respondents’ support for catch-and-release in the survey.

The questionnaire was designed based on a literature review of studies related to wallabies, including Drake et al. [10], APR Consultants [18], Booth [19], Grady et al. [20], Versus Research [21,22], Raftogianni et al. [23], and Leivers et al. [24], together with interviews with stakeholders, such as hunters, environmentalists, animal welfare advocates, and owners of private zoos housing wallabies (see Kaine [25] for more details). Given the importance of hunting in controlling wallaby numbers and supplying wallaby carcasses to meat processors and that hunters have the skills and knowledge to capture wallabies, we included questions about the respondents’ beliefs about (and experiences with) hunting in the questionnaire. The questionnaire is reproduced in Supplementary Materials Section S1.

Following the framework proposed by Kaine et al. [26], we hypothesised that respondents’ support for the eradication of wallabies would depend on their interest in (or enthusiasm for) the policy outcomes of protecting native birds and wildlife and protecting productive farmland, as well as their interest in (or enthusiasm for) the specific policy instrument of eradicating wallabies. The Kaine et al. [26] framework has been used to predict public support for the urban trapping of rats and possums and support for landscape-scale baiting programmes to control invasive pests [27,28,29].

A set of scales based on a condensed version of Laurent and Kapferer [30] was created to measure respondent interest in, or involvement with, protecting the environment, protecting farmland, and eradicating wallabies. Respondents indicated their agreement with statements representing each of the five components of involvement described by Laurent and Kapferer [30] using a 5-point rating that ranged from ‘strongly disagree’ (1) through to ‘strongly agree’ (5). For example, with respect to ‘protecting our native plants and wildlife’, the statements were about the following:

- Functional involvement concerned the importance of, and caring about, protecting our native plants and wildlife;
- Experiential involvement concerned the reward from, and passion about, protecting our native plants and wildlife;
- Identity-based involvement concerned opinions about protecting our native plants and wildlife reflecting on your identity, and others’ identity, as a person;
- Consequence-based involvement concerned the seriousness or importance of consequences arising from making a mistake in relation to protecting our native plants and wildlife;
- Risk-based involvement concerned making mistakes concerned the complexity or difficulty of making decisions about protecting our native plants and wildlife.

Similar statements were formulated for involvement with protecting productive farmland and with eradicating wallabies (see Supplementary Materials Section S1).

A second set of scales measured attitudes, as well as attitude strength, towards eradicating wallabies. Attitudes were measured using a simple, evaluative Likert scale. Theoretically, the strength of attitudes should vary depending on the strength of interest or involvement [31,32,33]. To test this hypothesis, respondents were also questioned about uncertainty in their attitude about eradicating wallabies using an ipsative, or ‘forced choice’, scale based on Olsen [34].

A third scale was designed to elicit the respondents’ potential level of support for catching-and-releasing wallabies. The seven statements in this scale were as follows:

- I am completely opposed to people catching-and-releasing wallabies back into the wild;
- I can understand why people rescue and release injured wallabies;
- I can understand why people catch-and-release baby wallabies;
- I can tolerate people catching-and-releasing wallabies for hunting;
- I completely support people catching-and-releasing wallabies back into the wild;
- I support people catching-and-releasing wallabies to create a food source;
- I support people catching-and-releasing wallabies to create jobs.

The strength of the respondents’ attitude towards catching-and-releasing wallabies was measured using an ipsative scale that was also based on Olsen [34].

A series of questions were formulated to discover respondent beliefs about the environmental, agricultural, and economic effects of wallabies, as well as their beliefs about eradicating wallabies. The ordering of the statements in the involvement, attitude scales, and the belief questions was randomised among the individual questionnaires to avoid introducing bias in responses. Respondents were questioned about their willingness to report seeing wallabies outside containment areas. Finally, respondents were questioned about their gender, age, education, income, and whether they had been hunting. Those that had been hunting were asked a series of additional questions about their hunting experiences.

Participation in the survey was voluntary: respondents could leave the survey at any time and all survey questions were optional and could be skipped. The questionnaire was approved for distribution by Manaaki Whenua—Landcare Research’s social ethics process (application 2223/28). The questionnaire was administered online by a commercial market research company. Respondents were randomly selected from a database of consumer panellists across New Zealand, stratified by regional population. The panellists received a small reward in the form of discount vouchers for completing surveys from the company maintaining the panel. The survey commenced in the second week of July 2023, and 1000 responses were received within a week.

Our analysis was guided by the following:

1. Following Kaine et al. [26], we hypothesised that respondent involvement with eradication would be positively correlated with involvement with protecting native plants and animals, as well as involvement with protecting farmland;
2. Following Ajzen and Fishbein [35] and Bagozzi [36], we hypothesised that respondent attitudes towards eradication would be correlated with beliefs about the effects of wallabies on the environment and farming, as well as with the degree to which wallaby numbers could be controlled through hunting;
3. Following Bagozzi [36], we hypothesised that respondent intentions to take action to achieve the behavioural goal of eradicating wallabies would be positively correlated with involvement with, and attitude towards, eradicating wallabies;
4. Given respondents’ need for consistency in their attitudes [37], we hypothesised that respondent support for catch-and-release would be negatively related to involvement with, and attitude towards, wallaby eradication;
5. Following Kaine et al. [26], we hypothesised that the strength of respondent attitudes towards eradicating wallabies, as well as the strength of support for catch-and-release, would be positively correlated with involvement with eradicating wallabies.

We tested hypotheses 1–4 using linear regression analysis. Hypothesis 5 was tested using multinomial logit regression analysis. Given the large number of belief variables, we condensed them into a small set of composite variables using principal components analysis with varimax rotation.

We also used linear regression analysis to conduct the following:

- Analyse the relationship between respondent willingness to report sightings of wallabies outside containment areas and involvement with, and attitude towards, eradicating wallabies;
- Analyse the relationship between respondents who were hunters and who supported catch-and-release and their hunting experiences.

The demographic representativeness of the sample was tested using chi-square goodness-of-fit tests. Statistical analyses were conducted using SPSS, version 27.0 [38].

## 3. Results

### 3.1. The Sample

Of the 1000 respondents, 47% were women. The age distribution of the sample was older than current New Zealand census estimates, and the proportion of respondents with a bachelor’s or postgraduate degree was substantially higher than the proportion in the New Zealand population (see Supplementary Materials Section S2). Households with incomes greater than New Zealand $50,000 were also substantially overrepresented in the sample. Correspondingly, households with incomes less than New Zealand $50,000 were underrepresented (see Supplementary Materials Section S2). All of these differences were statistically significant.

Statistical tests indicated that the involvement scales were reliable, that is, they were internally consistent in the sense that scores on related statements were highly correlated with one another (see Supplementary Materials Section S2).

Approximately 12 percent of respondents indicated that they were hunters. We did not find any statistically significant and meaningful differences between hunters and other respondents with respect to involvement with protecting native plants and animals, involvement with protecting farmland, involvement with eradicating wallabies, attitude towards eradicating wallabies, or support for catching-and-releasing wallabies. Meaningful was defined as Ɛ^2^ > 5% [39,40].

Approximately 6% of respondents had a strongly favourable attitude towards catching-and-releasing wallabies as they indicated that they strongly agreed that catching-and-releasing wallabies was the ’right thing to do’. Approximately 38% of respondents had a strongly unfavourable attitude towards catching-and-releasing wallabies as they indicated that they ‘strongly agreed’ that catching-and-releasing wallabies is the ’bad thing to do’.

### 3.2. Beliefs About Wallabies, Eradicating Wallabies, and Hunting

We used factor analysis to condense into a small set of composite belief variables of the respondents’ beliefs about the following: (1) the environmental, economic, and social effects of wallabies; (2) beliefs about eradicating wallabies; and (3) beliefs about hunting wallabies.

Respondent beliefs about the environmental, economic and social effects of wallabies were summarised by three composite variables (see Table 1). The first composite was interpreted as believing that wallabies have harmful environmental and economic effects and, consequently, should not be allowed in the wild in New Zealand. The second composite was interpreted as believing that wallabies create economic and recreational benefits. The third composite was interpreted as believing that, while wallabies harm the environment, they are not as damaging as other introduced species, such as pigs, possums and deer.

Respondent beliefs about eradicating wallabies were summarised by two composite variables (see Table 2). The first composite was interpreted as believing that at least some wallabies should be kept because they generate economic, recreational, and cultural benefits. The second composite was interpreted as believing that wallabies should be eradicated to protect native plants, wildlife, and agriculture.

Respondent beliefs about hunting wallabies were summarised by three composite variables (see Table 3). The first composite was interpreted as believing that hunting contributes to controlling wallaby numbers but is not always a practical means of doing so. The second composite was interpreted as believing that hunting is unsafe and can be a costly and inefficient means of controlling wallaby numbers. (Note that, depending on respondents’ interpretation of ‘cost effective’, this could mean that they believed that hunting wallabies was either personally costly or that hunting was more costly than other means of controlling wallabies, or perhaps both.) The third composite was interpreted as believing that recreational hunting is as practical and effective in controlling wallaby numbers as government programmes that use poison baits to control wallaby numbers.

### 3.3. Involvement in and Attitude Towards the Eradication of Wallabies

As hypothesised, respondent involvement (that is, interest) in the policy objective of eradicating wallabies depended on their involvement (interest) in protecting the native environment and their involvement (interest) in protecting farmland (see Table 4). Additionally, as hypothesised, respondent attitudes towards eradicating wallabies depended on (negative) beliefs about the need to keep wallabies and the need to eradicate them (see Table 4). These beliefs, in turn, depended on the respondents’ beliefs about the environmental, economic, and social effects of wallabies (see Table 4).

We hypothesised that respondent attitudes towards eradication were also influenced by beliefs about hunting wallabies, as these beliefs could shape views on the practicality of controlling or eradicating wallabies. This hypothesis was also supported with respondent attitudes towards eradication (together with their beliefs about the need to keep wallabies and the need to eradicate them) being influenced by beliefs about whether hunting helps control wallabies, whether hunting is unsafe and costly and whether hunting is equally as effective as government baiting programmes in controlling wallaby numbers (see Table 4).

### 3.4. Strength of Attitudes Towards the Eradication of Wallabies

The effects of beliefs about wallabies and hunting wallabies on the strength of respondent attitudes towards eradication were consistent with those reported above (see Table 5). The results supported the hypothesis that involvement with eradication, which depends on involvement with protecting native plants and wildlife and involvement with protecting farmland, distinguished respondents with a strongly favourable attitude towards eradication from those who were indifferent and those who had an unfavourable attitude towards eradication. Respondents who believed wallabies were harmful, that wallabies were just as harmful as other introduced species, and that wallabies did not create economic or social benefits, were more likely than those who thought otherwise to have a favourable attitude towards eradication.

Compared to respondents with a strongly unfavourable attitude towards eradication, respondents who were indifferent to (or ambivalent about) eradication, or who regarded it as personally irrelevant, were more likely to believe that wallabies were harmful, as harmful as other introduced species, and that wallabies did not create economic or social benefits (Table 5).

Respondents with a strongly favourable attitude towards eradicating wallabies were likely to believe that hunting helps to control wallaby numbers and that hunting is safe and practical but recreational hunting is not as effective in controlling wallaby numbers as government baiting programs. In contrast, respondents with a strongly unfavourable attitude towards eradication were likely to believe that hunting does not help control wallaby numbers, that recreational hunting is unsafe in that it is a danger to people and to livestock, and that recreational hunting is inefficient but is as effective in controlling wallaby numbers as government baiting programs (Table 5).

### 3.5. Involvement, Attitudes, and Goal Intentions

We measured respondent intentions to contribute to the behavioural goal of eradicating wallabies by their preparedness to take some responsibility, to act, to make sacrifices, and to work with others to eradicate wallabies. These intentions were primarily influenced by their involvement in and, to a lesser degree, their attitude towards eradication. Their beliefs about hunting had only a marginal, if any, influence on their intentions (see Table 6). Relatedly, involvement with eradicating wallabies, and attitude towards eradicating wallabies, influenced respondents’ willingness to report sightings of wallabies outside containment areas (see Supplementary Materials Section S3).

### 3.6. Support for Catch-and-Release of Wallabies

We had hypothesised that respondent support for catch-and-release was inversely related to their involvement with, and attitude towards, eradication. This hypothesis was supported with respect to the strength of respondents’ opposition to catch-and-release (see Table 7). However, while respondents’ attitudes towards eradication and their beliefs about hunting influenced their understanding of, and tolerance for, catching-and-releasing wallabies, their involvement did not (see Table 7).

The more strongly respondents believed that recreational hunting helps control wallaby numbers and that recreational hunting is as effective as government baiting programs in controlling wallaby numbers, the more likely they were to understand and tolerate the catch-and-release of wallabies (see Table 7). The more strongly respondents believed that recreational hunting was unsafe and costly, the more likely they were to understand and tolerate the catch-and-release of wallabies. Put another way, the more strongly respondents believed that recreational hunting was safe and cost-effective, the less likely they were to understand and tolerate the catch-and-release of wallabies (see Table 7). Relatedly, we found that that respondents who supported catch-and-release and who were hunters were more likely to wish there were more wallabies and that wallabies were more widespread to make hunting easier (see Supplementary Materials Section S4). The results regarding the strength of respondent support for catch-and-release are reported in Table 8. Involvement in, and attitude towards, eradicating wallabies distinguished respondents with strong attitudes from those who were indifferent, were ambivalent, or for those whom catch-and-release was personally irrelevant.

In Table 9, we report the correspondence between attitude strength regarding eradicating wallabies and attitude strength regarding the catch-and-release of wallabies. The results indicate consistency in the strength of the respondents’ attitudes.

The moderate-to-high involvement of most respondents (including hunters) with eradicating wallabies—as well as their favourable attitude towards eradicating wallabies—means that, to some degree, they will be willing to report sightings of wallabies outside containment areas (see Supplementary Materials Section S5).

## 4. Discussion

### 4.1. Rationale for Catch-and-Release

People’s preferences with respect to the management of a species depend, fundamentally, on their beliefs about the potential for the species to create environmental and economic benefits and harms [5,10,11,18,21,27,41,42]. Hence, the translocation of invasive species can be rationalised if one believes that the invasive species is not particularly harmful to the environment and that it is economically beneficial [10]. We found this to be the case regarding the illegal catch-and-release of wallabies.

The more strongly respondents believed that wallabies did not damage the environment and were economically beneficial, the more unfavourable their attitude was towards eradicating wallabies and the stronger their support for catch-and-release. By contrast, the more strongly respondents believed that wallabies did damage the environment and were not economically beneficial, the more favourable their attitude was towards eradicating wallabies and the stronger their opposition to catch-and-release.

Only a small minority of respondents, about 6 per cent, appeared to be strongly opposed to the eradication of wallabies and supportive of catch-and-release; most respondents appeared to be strongly supportive of eradication of wallabies and strongly opposed to catch-and-release. We also found that the strength of respondent attitudes towards eradication and catch-and-release were strongly influenced by their involvement with (that is, interest in) eradication, which—in turn—depended on their involvement with (interest in) protecting native plants and animals and protecting farmland.

We found that respondent attitudes towards eradication were strongly influenced by their beliefs about recreational hunting. Respondents who strongly favoured eradication were more likely to believe that hunting does not help control wallaby numbers compared to respondents who opposed eradication.

The desire to catch-and-release an invasive species for sport can also be rationalised by arguing that hunting contributes to the control of the species [23], although the extent to which hunting can contribute to the management of an invasive species is not always clear [19,43,44,45]. Other potentially salient factors are the authorised translocation of invasive species, such as trout, can also be viewed as setting an example for those contemplating illegal catch-and-release [46]; and the possibility, mentioned by some interviewees, that concerns for animal welfare (amongst animal rescuers, particularly) might motivate the illegal release of individual, rehabilitated wallabies.

We found that respondent attitudes towards catch-and-release were strongly influenced by beliefs about recreational hunting. Respondents who strongly supported catch-and-release were more likely to believe that hunting does help control wallaby numbers, that it is as effective in controlling wallaby numbers as baiting programs, but that it is unsafe and costly. These respondents are likely to have an unfavourable view of eradication because they believe that wallabies create economic benefits and do not severely damage the environment.

The support of these respondents for catch-and-release seems subjectively rational because they believe (1) wallabies create economic benefits, (2) that the damage wallabies inflict on the environment can be minimised through hunting and baiting programs, and (3) that if wallabies were more widespread hunting would be easier and safer (presumably, we speculated, because it would involve spending less time in remote areas with more rugged terrain). This reasoning was consistent with some interviewees who suggested hunters who might catch-and-release game animals may do so because they thought they could control the number and spread of the animals, as well as because they were not aware of the environmental risks associated with translocating animals (e.g., dissemination of diseases and soil-borne pathogens).

Conversely, respondents who strongly opposed catch-and-release were more likely to believe that hunting does not help control wallaby numbers and that hunting is not as effective in controlling wallaby numbers as are baiting programs, but recreational hunting is safe and cost effective. This suggests that these respondents see hunting as an inexpensive complement to baiting programs and that, presumably, eradication is achievable. Hence, these respondents tend to have a strongly unfavourable attitude towards catch-and-release because it jeopardises the goal of eradicating wallabies from New Zealand.

We found that the respondents who strongly support catch-and-release have a different attitude towards eradication than respondents who strongly oppose catch-and-release. This difference in attitudes was related to beliefs about the effects of wallabies and beliefs about hunting. We also found that respondents that support catch-and-release had relatively high involvement with eradicating wallabies, as did those who oppose it. In other words, respondents who support catch-and-release are, on average, as passionate about the subject of wallaby eradication as those that oppose catch-and-release. This means that respondents who support catch-and-release are, on average, as passionate about the subject of protecting native plants and animals (and farmland) as those that oppose catch-and-release. Hence, the differences in their attitude towards eradication and catch-and-release arise from the differences in their beliefs about the effects of wallabies and about hunting. Taken together, these two findings have important implications for designing interventions to reduce the frequency of catch-and-release and increase the effectiveness of efforts to eradicate wallabies from New Zealand.

### 4.2. Regulating Behaviour

Knopf and Dustin [47] termed behaviour ‘vandalistic’ when people engage in behaviours they know are illegal and have potentially damaging effects. They argued that harsh and highly visible sanctions, witness rewards, peer pressure, and enforcement are necessary to coerce these people into behaving responsibly. They suggest that sanctions should be severe and commensurate with the severity of potential ecological and socioeconomic impacts. The difficulty with their approach here is that enforcing sanctions and penalties is problematic because hunting mainly occurs in isolated areas making detection difficult and the chance of being caught low, while only a small number of animals may need to be released to do damage [48]. Logically, this implies that effective efforts to discourage the release of wallabies outside containment areas depend on policy instruments, such as incentives, promotions, and encouraging self-regulation [26]. However, we do note that the accidental release of wallabies from private zoos or petting farms is unlikely because these enterprises are tightly controlled through a licensing system that regulates the number of wallabies that can be kept, enclosure de-sign, and the breeding and sourcing of replacement stock [25].

### 4.3. Changing Behaviour by Changing Attitudes

High- and low-involvement behaviours are fundamentally different in terms of the cognitive effort that is invested in search for, and processing of, information prior to forming an attitude and making decisions [49]. Low involvement is associated with little, if any, search for information, as well as limited reasoning, which results in weak, unstable attitudes. High involvement leads to an extensive search for information and deliberate, thoughtful reasoning, resulting in strong, stable, and consistent attitudes that resist exogenous attempts to change them [31]. Consequently, changing the attitudes of those who strongly support catch-and-release will be challenging because their support is likely to be based on informed, thoughtful reasoning. In this regard, the illegal catch-and-release of wallabies is most similar, in terms of involvement and effort, to illegal hunting where hunters kill protected species or disrupt efforts to establish protected predators of game [8,50].

Changing their attitudes requires supplying credible evidence that may change beliefs about the effects of wallabies and their beliefs about hunting. This means supplying evidence (that the recipient judges find convincing) that wallabies are severely damaging to the environment and to the productivity of farmland. It also means providing convincing evidence that ground hunting alone cannot control wallaby numbers sufficiently to eradicate them at landscape scales and that hunting is not as effective as aerial baiting programs in controlling wallaby numbers. The implication is that illegal catch-and-release may eventually require introducing baiting programs to control them into new areas if wallabies become established.

Importantly, strong support for catch-and-release is associated with high involvement with protecting native plants and animals and with protecting farmland. This level of involvement means that those who strongly support catch-and-release are likely to notice, and consider the content of, promotional efforts highlighting the damaging effects wallabies can have on the native species and farm productivity. The same can be said, of course, for those that strongly oppose catch-and-release. The success of such efforts will depend on how compelling the recipient finds the evidence for the effects. Note that, because those who strongly support catch-and-release find wallaby eradication highly involving, it is unlikely that they are ignorant of the law regarding this behaviour [47].

### 4.4. Changing Behaviour by Modifying Social Norms

In principle, the frequency of catch-and-release may be reduced by establishing it as being inconsistent with other behaviours that supporters of catch-and-release find highly involving and attractive see [26]. For instance, as hunters appear to be the most likely potential source of wallabies for catch-and-release, their propensity to engage in this behaviour may be constrained if it is regarded by them as being inconsistent with ‘good’ hunting practice. In other words, catch-and-release is inconsistent with social norms regarding hunting behaviour [51]. However, the efficacy of this approach is likely to be limited as success is conditional on people engaging in the behaviour of interest being susceptible to the opinions of others who have an unfavourable attitude towards the behaviour (e.g., [10,52]) and who, therefore, lack source credibility; the more involving the behaviour, the more likely is it that people will engage in motivated reasoning [53] and discount the opinions of others who disagree with them.

Behavioural insights and nudges [54] are a related form of changing behaviour in that nudges often employ normative social information. However, nudges are unlikely to apply in the context of illegal catch-and-release because the behaviour is highly involving and invokes extensive decision making. Furthermore, catch-and-release is not a behaviour that others can easily observe [51]. Consequently, the behaviour is deliberate and does not rely on specific cognitive biases, boundaries, heuristics, and habits that nudges are designed to influence.

Fortunately, in this study, most hunters (like most respondents) had moderate-to-high involvement with protecting native plants and wildlife and with protecting farmland. They also had moderate involvement with eradicating wallabies, favourable attitudes towards eradicating wallabies, and unfavourable attitudes towards catch-and-release. This means that most hunters can be relied upon to self-regulate [26] and not release wallabies outside containment areas (or supply wallabies for release outside containment areas).

The moderate-to-high involvement of most respondents (including hunters) with eradicating wallabies—as well as their favourable attitude towards eradicating wallabies—means that, to some degree, they will be willing to report sightings of wallabies outside containment areas. This is an important finding because, in the absence of enforceable regulation, reports of sightings by hunters and the public are the only way in which the wallabies released outside containment areas might be rapidly detected.

### 4.5. Changing Behaviour Through Human-Centred Design

The general principles of human-centred design may be employed to change catch-and-release behaviour [51]. Human-centred design seeks to develop, refine, and implement a context-specific and locally suited behaviour change strategy by working directly and collaboratively with the individuals and communities affected [51]. The strategy relies on managing conflict constructively by anticipating disagreements, attending more carefully to the social–ecological contexts of management, adopting more inclusive engagement mechanisms, and fostering more open, responsive communication [55,56].

Examples of human-centred design that might influence catch-and-release behaviour contain incorporating recreational hunting into the management of wallabies, including the performance of sampling and monitoring tasks, as well as culling [45]; creating exclusive areas for recreational hunters; improving access to containment areas for recreational hunters (see Nugent et al. [57]); and removing restrictions on when hunting can occur and restrictions on bag limits. Another example, although not so relevant to wallaby hunting, would be the introduction of legislation that prohibits the charging of hunting fees to reduce the commercial incentive to catch-and-release game [43].

### 4.6. Limitations and Further Research

The extent to which support for catch-and-release translates into actual instances of this behaviour was not investigated. Hence, a limitation of the research is the extent to which support for catch-and-release translates into actual behaviour. Estimates of the frequency of such behaviour could be made using specialised questioning techniques that preserve the anonymity of respondents and, at least in principle, minimise social desirability bias (e.g., [8,58]. There may be merit in using these techniques in future studies, given the difficulty of obtaining actual data about catch-and-release.

The results clearly show that beliefs about hunting influence respondent attitudes towards wallaby eradication, support for catch-and-release, and willingness to report seeing wallabies in the wild. Research into the reasoning underpinning respondent beliefs about hunting wallabies, especially regarding its safety and its effectiveness relative to government baiting programmes, would assist in the design of promotional efforts aimed at discouraging the catch-and-release of wallabies.

The results also show that there were substantial differences among hunters in their beliefs about wallabies and, implicitly, substantial differences in their experiences hunting wallabies. Investigating these differences (and the reasons for them) would also be worthwhile to assist promotional efforts aimed at discouraging the catch-and-release of wallabies. Relatedly, the involvement of hunters (and others) with hunting was not investigated. Given the importance of involvement in choosing and designing interventions to change behaviour, research into involvement with hunting would be worthwhile.

Finally, we believe that there is value in investigating the differences between professional and recreational hunters. The interviewees were largely of the view that recreational hunters were the most likely group to engage in the illegal catch-and-release of wallabies. In principle, they may rescue and subsequently release young wallabies into the wild in the absence of an alternative means of disposing of them. While there are pet owners with low involvement who will abandon their pet due to callousness or boredom, for most pet owners, the decision to abandon their pet is a last resort forced upon them by circumstances [59], especially when it is illegal to keep a species as pets [60].

Devoted recreational hunters who hunt for the pleasure of exercising their skills and using specialist knowledge, such as bow hunters, can reasonably be expected to be highly involved with hunting. For these hunters, catching-and-releasing wallabies to reduce the effort entailed in hunting appears, on the surface at least, to be inconsistent with a key motivation for hunting (that is, the exercise of skill and specialist knowledge). There is the possibility that—if these hunters are travelling long distances to hunt and if there are suitably large areas of wallaby habitat in their region—they could be tempted to catch-and-release wallabies (and they probably have the skills and knowledge to do so successfully). Hence, there is value in understanding the differences between professional and recreational hunters to design appropriate interventions to influence their behaviour.

## 5. Conclusions

In New Zealand, Bennett’s and Tammar wallabies are damaging the native environment and agriculture, and their range is spreading. Efforts to reduce the threat they pose to the sustainability of native species biodiversity and agriculture are being put at risk by the illegal release of wallabies outside containment areas. We discovered that a very small proportion of the public supported the illegal catch-and-release of wallabies. We also found that the behaviour appeared to be motivated by a desire of a small proportion of recreational hunters to make the hunting of wallabies safer and easier by increasing their range. Respondents, including hunters, that supported catch-and-release believed that wallabies create economic benefits and do not severely damage the environment. They also believed that hunting helps control wallaby numbers and that it is as effective in controlling wallaby numbers as baiting programs. As these respondents believe that wallaby numbers can be controlled through hunting and baiting programs, their support for catch-and-release appears rational. These results highlight how vulnerable pest eradication programs can be opposition from a very small group of people.

## Figures and Tables

**Table 1:** Beliefs about wallabies.

Belief Statement	Wallabies Are Harmful	Wallabies Have Benefits	Wallabies Are Less Damaging than Other Pests
Wallabies damage our native plants and forests	0.80		
Wallabies damage orchards and gardens	0.77		
The harm caused by wallabies outweighs any benefits of having them roam free	0.77		
Wallabies are a danger to our native birds and wildlife	0.76		
Wallabies are a health risk to livestock	0.71		
Wallabies do not belong in New Zealand	0.70		
Wallabies compete with livestock for pasture	0.68		
Wallabies compete with deer for food sources	0.63		
Wallabies are a useful source of income for some people		0.80	
Wallabies are an important food source for some people		0.78	
Wallabies contribute to the economy by providing jobs for people		0.77	
Wallabies are important for recreational hunting		0.77	
Wallabies are a useful source of dog food		0.71	
Wallabies cause much less damage to the environment than wild pigs or possums			0.80
Wallabies cause much less damage to the environment than wild deer			0.79
Wallabies have just as much of a right to life as other animals	−0.40	0.34	0.49

Note: Factors represent 60% of the variance in the data. Values represent the correlation between the original variables and the three composite variables created in the factor analysis. Correlations of less than 0.30 are not reported, and the corresponding cells in the table are intentionally left blank.

**Table 2:** Beliefs about the need to eradicate wallabies.

Belief Statement	Need to Keep Some Wallabies	Need to Eradicate Wallabies
We need to have some wallabies to hunt for food	0.89	
We need to have some wallabies because they are a useful source of income for some people	0.88	
We need to have some wallabies for recreational hunting	0.87	
We need to have some wallabies because they are a useful source of dog food	0.87	
We need to have some wallabies for future generations to enjoy	0.83	−0.30
We need to have some wallabies for those who enjoy seeing wild animals	0.83	−0.35
Eradicating wallabies interferes with nature	0.72	
Wallabies have the right to exist wherever they may occur	0.72	−0.39
We need to eradicate wallabies to protect our native plants, birds and wildlife		0.86
We need to eradicate wallabies because they compete with livestock for pasture		0.85
Native species have greater rights than wallabies		0.76

Note: Factors represent 74% of the variance in the data. Values represent the correlation between the original variables and the three composite variables created in the factor analysis. Correlations of less than 0.30 are not reported, and the corresponding cells in the table are intentionally left blank.

**Table 3:** Beliefs about hunting.

Belief Statement	Hunting Helps Control Wallabies	Hunting Is Unsafe and Costly	Hunting Is Equally as Effective as Baiting Programmes
Hunting contributes to reducing and controlling wallaby numbers	0.79		
Recreational hunting supports government programmes to control wallaby numbers	0.76		
Hunting wallabies helps keep nature in balance	0.75		
Hunting wallabies helps control wildlife diseases	0.75		
Recreational hunting of wallabies is cost-free, so we may as well allow it	0.73		
Hunting is a more humane way to kill wallabies than poisoning	0.56		0.42
Hunting to reduce wallaby numbers is a risk to people’s health		0.80	
Hunting to reduce wallaby numbers is not cost-effective		0.80	
Hunting to reduce wallaby numbers is a danger to livestock		0.78	
Hunting to reduce wallaby numbers is not practical in some areas	0.30	0.69	−0.41
Recreational hunting is just as effective as government programmes in controlling wallabies	0.40		0.74
Hunting is just as effective as using poison baits to control wallaby numbers	0.33		0.73

Note: Factors represent 64% of the variance in the data. Values represent the correlation between the original variables and the three composite variables created in the factor analysis. Correlations less than 0.30 are not reported, and the corresponding cells in the table are intentionally left blank.

**Table 4:** Attitude towards eradication and nature of beliefs.

Variable	Involvement with Eradication	Attitude Towards Eradication	Need Some Wallabies	Need to Eradicate Wallabies
Involvement with protecting native plants and animals	0.359 ***			
Involvement with protecting farmland	0.345 ***			
Need some wallabies		−0.496 **		
Need to eradicate wallabies		0.613 **		
Wallabies are harmful			−0.197 ***	0.643 ***
Wallabies have benefits			0.512 ***	−0.012
Wallabies are relatively less damaging			0.254 ***	−0.181 ***
Hunting helps control wallabies		0.134 ***	0.065 **	0.176 ***
Hunting is unsafe and costly		0.027	0.255 ***	0.129 ***
Hunting is equally effective as government baiting programmes		−0.009	0.180 ***	0.052 *
Adjusted *R*^2^	0.39	0.70	0.66	0.58
*F*-test significance	<0.001	<0.001	<0.001	<0.001

Note: Values are standardised beta coefficients. n = 1000 for all regressions. * Indicates *p* < 0.05, ** indicates *p* < 0.01, and *** indicates *p* < 0.001.

**Table 5:** Beliefs and strength of attitude towards eradicating wallabies.

Variable	Support (Eradicating Wallabies Is the Right Thing to Do)	Indifferent (It Doesn’t Really Matter to Me)	Ambiguous (I Am Not Really Sure It’s the Best Way to Go)	Irrelevant (I Haven’t Put Much Thought into It)
Involvement with eradicating wallabies	35.392 ***	5.459 ***	4.367 ***	3.053 **
Wallabies are harmful	27.039 ***	7.815 ***	3.912 ***	7.881 ***
Wallabies have benefits	0.251 ***	0.276	0.528 *	0.354 **
Wallabies are less damaging	0.280 ***	0.510 *	0.629	0.402 **
Hunting helps control wallabies	2.847 ***	2.281 **	2.064 **	2.157 **
Hunting is unsafe and costly	0.354	2.018 **	1.309	1.231
Hunting is equally effective as government baiting programmes	0.024	0.735	0.791	0.752
Nagelkerke *R*^2^	0.64			
-2 Log Likelihood	1878.38			
Likelihood ratio significance	<0.001			

Note: Coefficients are likelihood ratios. n = 1000. Reference group had the following attitude: ‘I strongly believe that eradicating wallabies is a bad thing to do’. * Indicates *p* < 0.05, ** indicates *p* < 0.01, and *** indicates *p* < 0.001.

**Table 6:** The influence of involvement and attitudes on intentions to contribute to the goal of eradicating wallabies.

Variable	I Feel Some Responsibility for Helping to Eradicate Wallabies	I Am Prepared to Take Action to Help Eradicate Wallabies	I Am Prepared to Make Sacrifices to Help Eradicate Wallabies	I Think It Is Important to Work Together to Eradicate Wallabies
Involvement with eradication	0.666 ***	0.586 ***	0.607 ***	0.469 ***
Attitude towards eradication	0.117 ***	0.222 ***	0.184 ***	0.383 ***
Hunting helps control wallabies	0.002	0.062 **	0.042	0.070 **
Hunting is unsafe and costly	0.100 ***	0.114 ***	0.095 ***	0.057 **
Hunting is equally effective as government baiting programmes	0.070 **	0.025	0.036	0.003
Adjusted *R*^2^	0.59	0.62	0.59	0.63
*F*-Test significance	<0.001	<0.001	<0.001	<0.001

Note: Values are standardised beta coefficients. n = 1000 for all regressions. ** indicates *p* < 0.01, and *** indicates *p* < 0.001.

**Table 7:** Support for catching and releasing wallabies.

Variable	I Completely Support People Catching and Releasing Wallabies Back into the Wild	I Support People Catching and Releasing Wallabies to Create Jobs	I Can Tolerate People Catching and Releasing Wallabies for Hunting	I Support People Catching and Releasing Wallabies to Create a Food Source	I Can Understand Why People Might Release Full-Grown Pet Wallabies into the Wild	I Can Under-stand Why People Catch and Release Baby Wallabies	I Can Understand Why People Rescue and Release Injured Wallabies	I Am Completely Opposed to People Catching and Releasing Wallabies Back into the Wild
Involvement with eradication	0.056	0.068 *	−0.016	−0.029	0.016	0.016	0.021	0.250 ***
Attitude towards eradication	−0.540 ***	−0.466 ***	−0.321 ***	−0.361 ***	−0.335 ***	−0.335 ***	−0.418 ***	0.357 ***
Hunting helps control wallabies	0.025	0.112 ***	0.225 ***	0.181 ***	0.129 ***	0.129 ***	0.104 ***	0.080 **
Hunting is unsafe and costly	0.336 ***	0.314 ***	0.287 ***	0.326 ***	0.280 ***	0.280 ***	0.215 ***	0.051
Hunting is equally as effective government baiting programmes	0.215 ***	0.288 ***	0.327 ***	0.280 ***	0.170 ***	0.170 ***	0.132 ***	−0.148
Adjusted *R*^2^	0.53	0.47	0.37	0.38		0.25	0.26	0.34
*F*-test significance	<0.001	<0.001	<0.001	<0.001	<0.001	<0.001	<0.001	<0.001

Notes: Values are standardised beta coefficients. n = 1203 for all regressions. * Indicates *p* < 0.05, ** indicates *p* < 0.01, and *** indicates *p* < 0.001.

**Table 8:** Strength of support for catch-and-release.

Variable	Support (Catching and Releasing Wallabies Is the Right Thing to Do)	Indifferent (It Doesn’t Really Matter to Me)	Ambiguous (I Am Not Really Sure It’s the Best Way to Go)	Irrelevant (I Haven’t Put Much Thought into It)
Involvement with eradication	0.662	0.460 **	0.471 **	0.265 ***
Attitude towards eradication	0.138 ***	0.180 ***	0.268 ***	0.287 ***
Hunting helps control wallabies	1.442 *	1.447 *	0.988	0.955
Hunting is unsafe and costly	2.263 ***	3.590 ***	1.953 ***	1.638 ***
Hunting is equally effective as government baiting programmes	1.771 **	2.134 ***	1.464 ***	1.465 ***
Nagelkerke *R*^2^	0.46			
-2 Log Likelihood	2325.15			
Likelihood ratio significance	*p* < 0.001			

Note: Coefficients are likelihood ratios. n = 1000. Reference group had the following attitude: ‘I strongly believe that catching and releasing wallabies is a bad thing to do’. * Indicates *p* < 0.05, ** indicates *p* < 0.01, and *** indicates *p* < 0.001.

**Table 9:** Strength of support for catch-and-release and strength of support for eradication.

	Catch-and-Release				
Eradication	Catching and releasing wallabies is the right thing to do	It does not really matter to me	I am not really sure it’s the best way to go	I have not put much thought into it	Catching and releasing wallabies is a bad thing to do
Eradicating wallabies is the right thing to do	3.2	2.5	7.2	2.8	25.7
It does not really matter to me	0.8	8.3	4.9	1.7	0.2
I am not really sure it’s the best way to go	1.1	1.2	11.5	4.6	0.6
I have not put much thought into it	0.2	0.9	3.9	12.8	1.9
Eradicating wallabies is a bad thing to do	1.7	0.6	0.4	0.5	0.8
Total	7.0	13.5	27.9	22.4	29.2

Notes: Values are percentages of the sample. Χ^2^ = 877.9, *p* < 0.001.

## Data Availability

The dataset analysed in this study is available at https://osf.io/7nmcx/ (accessed on 10 September 2025).

## Supplementary Materials

The following supporting information can be downloaded at: https://www.mdpi.com/article/10.3390/ani15182700/s1, Section S1: Questionnaire; Section S2: Sample demographics; Section S3: Reliability of involvement scales; Section S4: Hunters and beliefs about hunting; Section S5: Willingness to report sightings. References [61,62,63] are cited in Supplementary Materials.
