# Supplementary material for: The Illegal Catch-and-Release of Wallabies
Source: Animals (Basel). 2025 Sep 15;15(18):2700. doi: 10.3390/ani15182700 (PMC12466825; doi:10.3390/ani15182700)
Supplement: Supplementary file 1 [file animals-15-02700-s001.zip › animals-3848380-supplementary.pdf]

Section S1: Questionnaire

Wallaby Survey 2023

Area

1) Firstly which of the following regions do you live in?

( ) Northland

( ) Auckland

( ) Waikato

( ) Bay of Plenty

( ) Gisborne

( ) Hawke's Bay

( ) Taranaki

( ) Manawatu-Whanganui

( ) Wellington

( ) Tasman/Nelson

( ) Marlborough

( ) West Coast

( ) Canterbury

( ) Otago

( ) Southland

Beliefs about wallabies

2) We are interested in your thoughts about the effects of eradicating wallabies in New Zealand. How strongly do you agree or disagree with the following statements?

	**Strongly agree**	**Agree**	**Unsure/neutral**	**Disagree**	**Strongly disagree**
Wallabies are a danger to our native birds and wildlife	(5)	(4)	(3)	(2)	(1)
Wallabies damage our native plants and forests	( )	( )	( )	( )	( )
Wallabies are important for recreational hunting	( )	( )	( )	( )	( )
Wallabies compete with deer for food sources	( )	( )	( )	( )	( )
Wallabies compete with livestock for pasture	( )	( )	( )	( )	( )
Wallabies damage orchards and gardens	( )	( )	( )	( )	( )
Wallabies have just as much of a right to life as other animals	( )	( )	( )	( )	( )

Wallabies are a health risk to livestock	( )	( )	( )	( )	( )
Wallabies are an important food source for some people	( )	( )	( )	( )	( )
Wallabies are a useful source of income for some people	( )	( )	( )	( )	( )
Wallabies cause much less damage to the environment than wild deer	( )	( )	( )	( )	( )
Wallabies cause much less damage to the environment than wild pigs or possums	( )	( )	( )	( )	( )
Wallabies are a useful source of dog food	( )	( )	( )	( )	( )
The harm caused by wallabies outweighs any benefits of having them roam free	( )	( )	( )	( )	( )
Wallabies do not belong in	( )	( )	( )	( )	( )

New Zealand					
Wallabies contribute to the economy by providing jobs for people	( )	( )	( )	( )	( )

Involvement with protecting native biodiversity.

3) How strongly do you agree or disagree with the following statements about conserving our native plants, birds and animals?

	**Strongly agree**	**Agree**	**Unsure/neutral**	**Disagree**	**Strongly disagree**
I think protecting our native plants and wildlife is rewarding	( )	( )	( )	( )	( )
The consequences are serious if we fail to protect our native plants and wildlife	( )	( )	( )	( )	( )
Protecting our native plants and wildlife is something I am passionate about	( )	( )	( )	( )	( )
It would be a big deal if we failed to protect our native plants and wildlife	( )	( )	( )	( )	( )

My position about protecting our native plants and wildlife tells others something about me	( )	( )	( )	( )	( )
Protecting our native plants and wildlife is important to me	( )	( )	( )	( )	( )
Making decisions about protecting our native plants and wildlife is complicated	( )	( )	( )	( )	( )
What others think about protecting our native plants and wildlife tells me something about them	( )	( )	( )	( )	( )
I care a lot about protecting our native plants and wildlife	( )	( )	( )	( )	( )
Making decisions about protecting our native plants and wildlife is difficult	( )	( )	( )	( )	( )

Taking responsibility and action for biodiversity.

4) We are interested in how strongly you feel about the need for action to be taken to protect and conserve native biodiversity. How strongly do you agree or disagree with the following statements?

	**Strongly agree**	**Agree**	**Unsure/neutral**	**Disagree**	**Strongly disagree**
I think protecting our native plants and wildlife is the right thing to do	( )	( )	( )	( )	( )
I feel some responsibility for helping protect our native plants and wildlife	( )	( )	( )	( )	( )
I am prepared to act to protect our native plants and wildlife	( )	( )	( )	( )	( )
It is important to work together to protect our native plants and wildlife	( )	( )	( )	( )	( )
I am prepared to make sacrifices to protect our native plants and wildlife	( )	( )	( )	( )	( )

Involvement with protecting productive farmland.

5) How strongly do you agree or disagree with the following statements about protecting our productive farmland?

	**Strongly agree**	**Agree**	**Unsure/neutral**	**Disagree**	**Strongly disagree**
I think protecting our farmland is rewarding	( )	( )	( )	( )	( )
The consequences are serious if we fail to protect our farmland	( )	( )	( )	( )	( )
Protecting our farmland is something I am passionate about	( )	( )	( )	( )	( )
It would be a big deal if we failed to protect our farmland	( )	( )	( )	( )	( )
My position about protecting our farmland tells others something about me	( )	( )	( )	( )	( )
Protecting our farmland is important to me	( )	( )	( )	( )	( )
Making decisions about protecting our farmland is complicated	( )	( )	( )	( )	( )

What others think about protecting our farmland tells me something about them	( )	( )	( )	( )	( )
I care a lot about protecting our farmland	( )	( )	( )	( )	( )
Making decisions about protecting our farmland is difficult	( )	( )	( )	( )	( )

Taking responsibility and action for farmland.

6) We are interested in how strongly you feel about the need for action to be taken to protect farmland. How strongly do you agree or disagree with the following statements?

	**Strongly agree**	**Agree**	**Unsure/neutral**	**Disagree**	**Strongly disagree**
I think protecting our farmland is the right thing to do	( )	( )	( )	( )	( )
I feel some responsibility for helping protect our farmland	( )	( )	( )	( )	( )
I am prepared to make sacrifices to protect our farmland	( )	( )	( )	( )	( )
It is important to	( )	( )	( )	( )	( )

work together to protect our farmland					

Beliefs about eradicating wallabies

7) We are interested in your thoughts about eradicating wallabies. How strongly do you agree or disagree with the following statements?

	**Strongly agree**	**Agree**	**Unsure/neutral**	**Disagree**	**Strongly disagree**
We need to eradicate wallabies to protect our native plants, birds and wildlife	( )	( )	( )	( )	( )
We need to have some wallabies for recreational hunting	( )	( )	( )	( )	( )
We need to eradicate wallabies because they compete with livestock for pasture	( )	( )	( )	( )	( )
Wallabies have the right to exist wherever they may occur	( )	( )	( )	( )	( )
We need to have some	( )	( )	( )	( )	( )

wallabies to hunt for food					
We need to have some wallabies for those who enjoy seeing wild animals	( )	( )	( )	( )	( )
We need to have some wallabies for future generations to enjoy	( )	( )	( )	( )	( )
We need to have some wallabies because they are a useful source of income for some people	( )	( )	( )	( )	( )
Native species have greater rights than wallabies	( )	( )	( )	( )	( )
We need to have some wallabies because they are a useful source of dog food	( )	( )	( )	( )	( )
Eradicating wallabies interferes with nature	( )	( )	( )	( )	( )

Involvement with a program for eradicating wallabies

8) How strongly do you agree or disagree with the following statements about eradicating wallabies?

	**Strongly agree**	**Agree**	**Unsure/neutral**	**Disagree**	**Strongly disagree**
I think helping to eradicate wallabies would be rewarding	( )	( )	( )	( )	( )
The consequences are serious if we fail to eradicate wallabies	( )	( )	( )	( )	( )
Eradicating wallabies is something I am passionate about	( )	( )	( )	( )	( )
It would be a big deal if we failed to eradicate wallabies	( )	( )	( )	( )	( )
My position about eradicating wallabies tells others something about me	( )	( )	( )	( )	( )
Eradicating wallabies is important to me	( )	( )	( )	( )	( )
Making decisions about eradicating wallabies is complicated	( )	( )	( )	( )	( )

What others think about eradicating wallabies tells me something about them	( )	( )	( )	( )	( )
I care a lot about eradicating wallabies	( )	( )	( )	( )	( )
Making decisions about eradicating wallabies is difficult	( )	( )	( )	( )	( )

Taking responsibility and action for eradicating wallabies.

9) We are interested in how strongly you feel about the need for action to be taken to eradicate wallabies. How strongly do you agree or disagree with the following statements?

	**Strongly agree**	**Agree**	**Unsure/neutral**	**Disagree**	**Strongly disagree**
I feel some responsibility for helping to eradicate wallabies	( )	( )	( )	( )	( )
I am prepared to take action to help eradicate wallabies	( )	( )	( )	( )	( )
It is important to work together to eradicate wallabies	( )	( )	( )	( )	( )

I am prepared to make sacrifices to help eradicate wallabies	( )	( )	( )	( )	( )

Attitude towards eradicating wallabies

10) How strongly do you agree or disagree with the following statements about eradicating wallabies?

	**Strongly agree**	**Agree**	**Unsure/neutral**	**Disagree**	**Strongly disagree**
I think we should eradicate wallabies	( )	( )	( )	( )	( )
I think eradicating wallabies is the right thing to do	( )	( )	( )	( )	( )
I believe it is wrong to eradicate wallabies	( )	( )	( )	( )	( )

11) Which of the following statements best describes you?

(5) I really think eradicating wallabies is the right thing to do

(4) It doesn’t really matter to me whether wallabies are eradicated or not

(3) I am not sure if eradicating wallabies is the best way to go

(2) I haven’t put much thought into eradicating wallabies

(1) I strongly believe that eradicating wallabies is a bad thing to do

Thoughts on catch-and-release.

12) How strongly do you agree or disagree with the following statements about people catching and releasing wallabies into the wild?

	Strongly agree	Agree	Unsure/neutral	Disagree	Strongly disagree
I am completely opposed to people catching and releasing wallabies back into the wild	( )	( )	( )	( )	( )
I can understand why people rescue and release injured wallabies	( )	( )	( )	( )	( )
I can understand why people catch and release baby wallabies	( )	( )	( )	( )	( )
I can tolerate people catching and releasing wallabies for hunting	( )	( )	( )	( )	( )
I completely support people catching and releasing wallabies	( )	( )	( )	( )	( )

back into the wild					
I support people catching and releasing wallabies to create a food source	( )	( )	( )	( )	( )
I support people catching and releasing wallabies to create jobs	( )	( )	( )	( )	( )

13) Which of the following statements best describes you?

(5) I think catching and releasing wallabies is the right thing to do

(4) It doesn't really matter to me whether people catch and release wallabies or not

(3) I am not really sure if catching and releasing wallabies is the best way to go

(2) I haven't put much thought into people catching and releasing wallabies

(1) I strongly believe that catching and releasing wallabies is a bad thing to do

---

Perceived effects of hunting on managing wallaby numbers.

14) How strongly do you agree or disagree with the following statements about relying on hunting to reduce wallaby numbers?

	**Strongly agree**	**Agree**	**Unsure/neutral**	**Disagree**	**Strongly disagree**
Hunting to reduce wallaby numbers is not cost effective	( )	( )	( )	( )	( )

Hunting to reduce wallaby numbers is not practical in some areas	( )	( )	( )	( )	( )
Hunting to reduce wallaby numbers is a risk to people's health	( )	( )	( )	( )	( )
Hunting contributes to reducing and controlling wallaby numbers	( )	( )	( )	( )	( )
Recreational hunting supports government programs to control wallaby numbers	( )	( )	( )	( )	( )
Hunting is a more humane way to kill wallabies than poisoning	( )	( )	( )	( )	( )
Hunting to reduce wallaby numbers is a danger to livestock	( )	( )	( )	( )	( )
Hunting is just as effective as using poison baits to control	( )	( )	( )	( )	( )

wallaby numbers					
Recreational hunting is just as effective as government programs in controlling wallabies	( )	( )	( )	( )	( )
Recreational hunting of wallabies is cost-free, so we may as well allow it	( )	( )	( )	( )	( )
Hunting wallabies helps keep nature in balance	( )	( )	( )	( )	( )
Hunting wallabies helps control wildlife diseases	( )	( )	( )	( )	( )

Hunting

*15) Do you go hunting?**

(1) Yes

(0) No

Experiences with hunting wallabies

16) How strongly do you agree or disagree with the following statements about hunting wallabies?

	**Strongly agree**	**Agree**	**Unsure/neutral**	**Disagree**	**Strongly disagree**
Hunting wallabies is fun	( )	( )	( )	( )	( )
Hunting wallabies takes a lot of skill	( )	( )	( )	( )	( )
I wish there were more wallabies so hunting would be easier	( )	( )	( )	( )	( )
Hunting wallabies is challenging because they behave differently to other game animals	( )	( )	( )	( )	( )
I am encouraged to keep hunting when I do shoot a wallaby	( )	( )	( )	( )	( )
I get excited when I hunt wallabies	( )	( )	( )	( )	( )
I think the idea that hunting makes a difference to wallaby numbers is naive	( )	( )	( )	( )	( )

I wish wallabies had a bigger range, so I don't have to travel so far to hunt them	( )	( )	( )	( )	( )

16b) How strongly do you agree or disagree with the following statements about hunting wallabies?

By hunting wallabies, I am setting a good example for my friends and family	( )	( )	( )	( )	( )
I feel like I am making a difference when I hunt wallabies	( )	( )	( )	( )	( )
I think hunting wallabies is useful	( )	( )	( )	( )	( )
Hunting is a practical way to reduce wallaby numbers	( )	( )	( )	( )	( )
Hunting wallabies is helpful	( )	( )	( )	( )	( )
Distinguishing males from females is difficult with wallabies	( )	( )	( )	( )	( )
If I can, I prefer to hunt male wallabies	( )	( )	( )	( )	( )

It's getting harder to find wallabies in the wild	( )	( )	( )	( )	( )
Hunting wallabies is a practical way of getting game meat	( )	( )	( )	( )	( )
I prefer hunting deer, pigs or possums rather than hunting wallabies	( )	( )	( )	( )	( )

Attitude towards reporting wallabies

17) How strongly do you agree or disagree with the following statements about reporting wallabies?

	**Strongly agree**	**Agree**	**Unsure/neutral**	**Disagree**	**Strongly disagree**
I think we should report seeing wallabies or signs of them	( )	( )	( )	( )	( )
I think reporting seeing wallabies or signs of them is the right thing to do	( )	( )	( )	( )	( )
I believe it is wrong to	( )	( )	( )	( )	( )

report seeing wallabies or signs of them					

18) Which of the following statements best describes you?

(5) I think reporting seeing wallabies or signs of them is the right thing to do

(4) It doesn’t really matter to me whether people do or don’t report seeing wallabies or signs of them

(3) I am not sure having people report seeing wallabies or signs of them is the best way to go

(2) I haven’t put much thought into people reporting seeing wallabies or signs of them

(1) I strongly believe that having people report seeing wallabies or signs of them is a bad thing to do

---

Wallabies in New Zealand

19) Do you know if there are wallabies in the wild in New Zealand?

(1) Yes

(0) No

(3) Unsure

Wallabies in the wild

20) Have you ever seen a wallaby in the wild?

( ) Yes

( ) No

( ) Unsure

21) Have you ever seen signs (droppings, footprints) of a wallaby in the wild?

( ) Yes

( ) No

( ) Unsure

Reporting wallabies in the wild

22) Would you report seeing a wallaby (or signs of one) in the wild to your Regional Council, Biosecurity New Zealand, or the Department of Conservation?

( ) Yes

( ) No

( ) Unsure

23) Do you know how to report seeing a wallaby (or signs of one) in the wild to your Regional Council, Biosecurity New Zealand, or the Department of Conservation?

( ) Yes

( ) No

( ) Unsure

24) Have you ever reported seeing a wallaby (or signs of one) in the wild to your Regional Council, Biosecurity New Zealand, or the Department of Conservation?

( ) Yes

( ) No

25) How easy was it to report seeing a wallaby (or signs of one) in the wild to your Regional Council, Biosecurity New Zealand, or the Department of Conservation?

(5) Very easy

(4) Fairly easy

(3) Okay

(2) Fairly difficult

(1) Very difficult

26) If you were to kill a wallaby in the wild, how willing would you be to report the kill anonymously on the Biosecurity New Zealand website?

(5) Very willing

(4) Willing

(2) Unwilling

(1) Very unwilling

(3) Don't know

Demographics

27) We just have a few questions to make sure we get a good cross-section of people. What age bracket do you fit into?

( ) 18 to 29

( ) 30 to 39

( ) 40 to 49

( ) 50 to 59

( ) 60 to 69

( ) 70 and over

( ) Prefer not to say

---

28) Which of the following do you identify as?

(1) Male

(2) Female

(3) Gender diverse

(0) Prefer not to say

---

29) What is your highest level of formal education?

( ) Some or all of secondary school

( ) Certificate (1-6)

( ) Diploma (5-7)

( ) Bachelor degree

( ) Post-graduate diploma/certificate

( ) Post-graduate degree

( ) Prefer not to say

---

30) What household income bracket do you fit into?

( ) Less than $20,000

( ) $20,000 to $50,000

( ) 50,000 to $75,000

( ) $75,000 to $100,000

( ) More than $100,000

( ) Prefer not to say

---

31) Are you a member of any of the following groups?

[ ] Hunting association

[ ] Environment association

[ ] Animal rights group

[ ] Animal rescue group

[ ] Farming association

[ ] Other - Write In (Required): ____

[ ] Prefer not to say

---

32) Do you live in?

( ) A large city

( ) A provincial centre

( ) A small country town

( ) The countryside

( ) Prefer not to say

33) Please include your occupation below.

____

---

34) That is the end of the survey.

Do you have any thoughts about wallabies you would like to share with us?

____

____

____

____

Thank You!

Section S2: Sample demographics

Table S2.1. Age distribution of respondents

Age category (years)	Percentage of respondents	Percentage of New Zealand residents
18–29	21.5	25.5
30–39	22.7	16.2
40–49	18.7	16.2
50–59	16.8	16.2
60–69	11.8	13.0
70 and over	8.5	12.9

Source: [61]

Table S2.2. Distribution of respondents by highest educational qualification

Education category	Percentage of respondents	Percentage of New Zealand residents
Some or all of secondary school	13.1	16.6
Certificate (1–5)	17.0	44.1
Diploma (6–7)	14.0	10.5
Bachelor's degree	27.7	16.5
Graduate or postgraduate degree	25.4	12.3

Source: [61]

Table S2.3. Income distribution of respondents

Income category	Percentage of respondents	Approximate percentage of New Zealand households
Less than $20,000	2.7	20.0
$20,000 to $50,000	14.5	50.0
$50,000 to $75,000	14.8	20.0
More than $75,000	50.7	20.0

Notes: Based on household disposable income deciles. First decile <$23,530, second to sixth deciles $23,530 to $54,665, seventh and eighth deciles <$54,665 to $72,895, remaining deciles >$72,895.

Source: [62]

Section S3: Reliability of involvement scales

Table S3.1. Reliability of involvement and attitude scales

Involvement or attitude	Reliability coefficient
Involvement with protecting native plants and wildlife	0.853
Involvement with protecting farmland	0.883
Involvement with eradicating wallabies	0.861
Attitude towards eradication	0.857
Support for catch-and-release	0.886

Notes: Reliability coefficient is Cronbach's alpha [63].

Section S4: Hunters and beliefs about hunting

Table S4.1. Selected correlations for respondents who hunted

Involvement or attitude	I wish there were more wallabies so hunting would be easier	I wish wallabies had a bigger range, so I don't have to travel so far to hunt them
Hunting helps control wallabies	-0.20*	-0.21*
Hunting is unsafe and costly	0.53**	0.51**
Hunting is equally as effective as baiting programmes	0.31	0.31**
Attitude towards eradication	-0.50**	-0.57**
Support for catch-and-release	0.74	0.66

Notes: Values are Pearson correlation coefficients. n=120 which is the number of respondents indicating that they went hunting. * Indicates P <0.05, ** indicates P <0.01, *** indicates P <0.001.

Section S5: Willingness to report sightings

Table S5.1. The influence of involvement and attitudes on intentions to report seeing wallabies

Variable	Intend to report seeing wallabies	Might report seeing wallabies
Involvement with eradication	2.828***	0.994
Attitude towards eradication	3.482***	2.115***
Hunting helps control wallabies	0.835	0.879
Hunting is unsafe and costly	0.439***	0.540***
Hunting is equally effective as government baiting programmes	0.697**	0.753*
Nagelkerke R^2	0.34	
-2 Log Likelihood	1518.15	
Likelihood ratio significance	$P < 0.001$	

Note: Coefficients are likelihood ratios. n =925. Reference group is having the attitude: 'I strongly believe that catching and releasing wallabies is a bad thing to do'. * Indicates P <0.05, ** indicates P <0.01, *** indicates P <0.001.
